# Can Simulation Measure Differences in Task-Switching Ability Between Junior and Senior Emergency Medicine Residents?

**DOI:** 10.5811/westjem.2015.12.28269

**Published:** 2016-02-10

**Authors:** Dustin Smith, Daniel G. Miller, Jeffrey Cukor

**Affiliations:** * Loma Linda University, Department of Emergency Medicine, Loma Linda, California; † University of Iowa, Department of Emergency Medicine, Iowa City, Iowa; ‡ University of Massachusetts, Department of Emergency Medicine, Worcester, Massachusetts

## Abstract

**Introduction:**

Work interruptions during patient care have been correlated with error. Task-switching is identified by the Accreditation Council for Graduate Medical Education (ACGME) as a core competency for emergency medicine (EM). Simulation has been suggested as a means of assessing EM core competencies. We assumed that senior EM residents had better task-switching abilities than junior EM residents. We hypothesized that this difference could be measured by observing the execution of patient care tasks in the simulation environment when a patient with a ST-elevation myocardial infarction (STEMI) interrupted the ongoing management of a septic shock case.

**Methods:**

This was a multi-site, prospective, observational, cohort study. The study population consisted of a convenience sample of EM residents in their first three years of training. Each subject performed a standardized simulated encounter by evaluating and treating a patient in septic shock. At a predetermined point in every sepsis case, the subject was given a STEMI electrocardiogram (ECG) for a separate chest pain patient in triage and required to verbalize an interpretation and action. We scored learner performance using a dichotomous checklist of critical actions covering sepsis care, ECG interpretation and triaging of the STEMI patient.

**Results:**

Ninety-one subjects participated (30 postgraduate year [PGY]1s, 32 PGY2s, and 29 PGY3s). Of those, 87 properly managed the patient with septic shock (90.0% PGY1s, 100% PGY2, 96.6% PGY 3s; p=0.22). Of the 87 who successfully managed the septic shock, 80 correctly identified STEMI on the simulated STEMI patient (86.7% PGY1s, 96.9% PGY2s, 93.1% PGY3s; p=0.35). Of the 80 who successfully managed the septic shock patient and correctly identified the STEMI, 79 provided appropriate interventions for the STEMI patient (73.3% PGY1s, 93.8% PGY2s, 93.8% PGY3s; p=0.07).

**Conclusion:**

When management of a septic shock patient was interrupted with a STEMI ECG in a simulated environment we were unable to measure a significant difference in the ability of EM residents to successfully task-switch when compared across PGY levels of training. This study may help refine the use of simulation to assess EM resident competencies.

## INTRODUCTION

Interruption of physicians during task performance has been well documented.^1^ These distractions to patient care occur more frequently in the emergency department (ED) than in outpatient settings.^2^ Both an increase in time-to-task completion and failure to return to task are correlated with interruptions.^3^ Previous studies have observed a difference in the ability to manage a simulated patient when comparing between participants’ level of experience.^4^,^5^ Task-switching is identified as a patient care competency in the Accreditation Council for Graduate Medical Education (ACGME) Next Accreditation System Milestones project.^6^ Simulation has been proposed as a method of assessing these milestones.^7^ We assumed that level of training affects emergency medicine (EM) resident physicians’ ability to execute required patient care tasks and hypothesized that this effect could be measured in the simulation environment when the care of a septic shock patient was interrupted by a second patient with an ST-elevation myocardial infarction (STEMI).

## METHODS

We performed a multicenter, prospective, observational cohort study on a convenience sample of residents in their first three years of training at three ACGME-accredited EM residency programs. At each site all eligible residents were enrolled. Participating sites included Loma Linda University Medical Center, UMass Memorial Medical Center and The University of New Mexico Hospital. Sites were selected during a Medical Education Research Certificate Program session at a Council of EM Residency Directors meeting by virtue of having a pre-existing simulation program and faculty members interested in measuring resident task-switching abilities. Institutional review board approval was obtained at each of the participating sites. Data collection occurred in the spring at all sites. Prior to data collection, residents at each of the study sites regularly participated in simulation as part of their residency curriculum, and all sites had previously covered sepsis and STEMI in didactic educational sessions. The three investigators collaborated to develop an immersive simulated patient encounter with the primary objective being the application of task-switching in order to address a time-sensitive distraction while caring for a critically ill patient. Prior to being used with study subjects each investigator piloted the simulated patient encounter and the data collection sheet by observing American Board of EM–eligible attending physicians managing the simulation. Subsequently, issues with the simulated case were resolved by discussion and consensus among the principal investigators. We assessed for baseline differences in electrocardiogram (ECG) interpretation skills by having each subject provide written interpretations to a series of ECGs in the weeks prior to the simulation testing. The STEMI ECG used in the simulation testing was incorporated among the ECGs for the written test, and each subject’s interpretation of the STEMI ECG was recorded for later analysis. This allowed subjects to serve as their own controls with respect to ECG interpretation abilities.

For the simulations, all sites used a high-fidelity mannequin in an environment that closely resembled an ED patient care area by having typical equipment, personnel, and systems found in the ED present for the simulation activity. The three investigators administered the simulation using a script with predetermined verbal responses and physiologic changes to interventions. Each study subject was presented with a 61-year-old diabetic male complaining of cough, fever, and shortness of breath with initial vital signs consistent with sepsis. Chest radiograph and physical exam findings were consistent with right lobar pneumonia. In each case the septic patient became hypotensive immediately after the chest radiograph was interpreted. At this point in the simulation each study subject was given a STEMI ECG of a separate chest pain patient presenting to triage and asked by the ECG technician for an interpretation and next action. Using a standardized data collection sheet with dichotomized responses, data were collected on the subjects’ treatment of the septic shock patient and on recognition and treatment of the STEMI patient. Acceptable interventions for the septic shock patient were defined as administration of appropriate empiric antibiotics, intravenous (IV) fluids, and pressors per early goal-directed therapy standards. Acceptable interventions for the STEMI patient were defined as performing any of the following actions after being given the STEMI ECG: activation of the cardiac catheterization lab, verbalizing their intent to personally evaluate the STEMI patient immediately, or verbalizing a request for another physician to evaluate the STEMI patient immediately.

We used the Cochran-Mantel-Haenszel contingency table analysis to analyze whether the number of years of training predicted the ability of a resident to properly manage sepsis while being presented with the STEMI ECG. We used logistic regression to analyze if the number of years of training predicts the ability of a resident to properly interpret a STEMI ECG while managing a septic shock patient.

## RESULTS

Ninety-one subjects participated: 30 post-graduate year (PGY)1s, 32 PGY2s, and 29 PGY3s. Eighty-seven subjects properly managed the patient with septic shock: 90.0% PGY1s, 100% PGY2s, and 96.6% PGY3s (p=0.22). Four subjects did not properly manage the patient with septic shock. One PGY1 did not appropriately order IV fluids and three PGY1s and one PGY3 did not appropriately order antibiotics.

Of the 87 who successfully managed the septic shock, 80 correctly identified STEMI on the simulated STEMI patient (86.7% PGY1s, 96.9% PGY2s, and 93.1% PGY3s; p=0.35). Of the 80 who successfully managed the septic shock patient and correctly identified STEMI on the simulated STEMI patient, 79 provided appropriate interventions for the STEMI patient (73.3% PGY1s, 93.8% PGY2s, 93.8% PGY3s; p=0.07). Both of the subjects who failed to provide appropriate interventions for the simulated STEMI patient correctly identified the STEMI in the simulation session but failed to identify the STEMI in the written test given prior to the simulation session. The Table and Figure provide resident testing characteristics by number of years of training.

## DISCUSSION

When we used simulation to measure EM resident physicians’ ability to task-switch between management of septic shock, and responding to a STEMI ECG, we observed no statistically significant difference between groups with different years of EM residency training.

We considered two possible interpretations of this data: (1) EM residents acquire the task-switching skills needed to concurrently execute sepsis and STEMI-related patient care tasks during their first year of EM residency; (2) our simulation scenario lacked the discriminatory power needed to measure task-switching ability differences between different years of EM residency training.

The first proposed interpretation contradicts prior research showing that resident year of training and EM residents’ scores on a multi-tasking assessment tool can explain variability in resident work efficiency.^8^ It also contradicts the authors’ anecdotal experiences after more than 20 collective years of supervising EM residents. Expert performance is mediated in part by highly structured and richly interconnected domain-specific knowledge.^9^ Dynamic decisions are real-time decisions that are interdependent and highly constrained by the decision-making environment.^10^

The second proposed interpretation of our data seems more in accord with the authors’ experiences and the aforementioned literature. The EM Milestone Project lists a series of multi-tasking (task-switching) milestones (subcompetency number 8) that residents are expected to achieve during training. Our data failed to show a difference between junior and senior EM residents in regards to ability to task-switch between different patients–a level 2 milestone.^6^ It seems most plausible that our scenario was unable to differentiate resident physicians’ abilities to manage patients amidst distraction because the simulation scenario did not require behaviors described in the level 3–5 milestones.

## LIMITATIONS

Our study has several limitations: (1) Inter-rater reliability was not assessed; (2) the study was performed in the simulation environment so the conclusions may not be applicable to the ED; (3) the number of residents who did not successfully perform the critical actions required for the patient in septic shock or the patient with a STEMI was small. Assuming that 60% of PGY1s would manage both patients correctly, our study had an 80% power to detect a 29% difference between PGY1 and PGY2-3s ability to manage both patients correctly. Because 73% of PGY1s managed both cases correctly, more than 100% of PGY2-3s would have to manage the case correctly in order for our study to measure a difference, the impossibility of this highlights the main limitation of our study–that the cases were not difficult enough to differentiate learners’ abilities. It is unknown how many distractions per hour would be needed to see a difference in residents at different levels of training if such a difference exists. Interestingly, while not obtained in the data collection tool, each researcher observed that with the less experienced subjects there appeared to be a trend towards a longer time period between verbalization of the ECG as a STEMI and performing appropriate interventions. It is possible that a time-to-action metric may have captured these differences; this would correlate with multi-tasking milestone level 3. This may be an additional area for future fruitful research as studies in other domains have demonstrated decreased decision-making performance by individuals performing dynamic tasks when under time constraints compared to static task performance under time constraints.^11^,^12^

## CONCLUSION

When management of septic shock was interrupted with a STEMI ECG in a simulation environment we observed no significant difference in completion of sepsis therapy or treatment of STEMI when compared between years of training. In our study 73% of PGY1s effectively task-switched between patients, while 94% of PGY2s and PGY3s demonstrated this ability. This observation supports the expectation that most EM residents achieve level 2 of the task-switching milestone by the end of their PGY1 year and nearly all should achieve level 2 by the end of their PGY2 year. This study suggests that future attempts to use simulation to measure differences in EM resident abilities, when comparing years of training, must incorporate skills at or above the level 3 milestone descriptors.

## Figures and Tables

**Figure:**
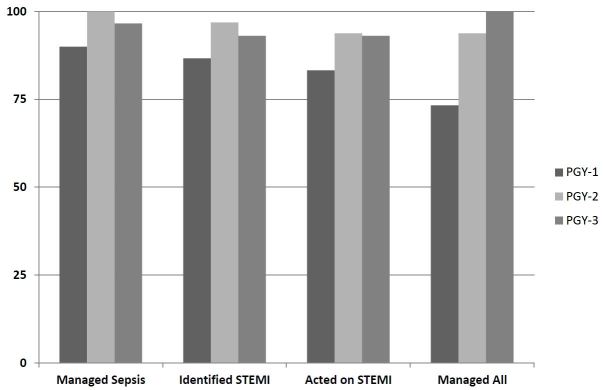
Resident performance on distraction study tasks by years of training. *PGY*, post graduate year; *STEMI*, ST segment elevation myocardial infarction

**Table:** Resident testing characteristics by number of years of training (n=91).

	PGY-1 (%)	PGY-2 (%)	PGY-3 (%)	Total (%)	P-value^a^
Correctly identify STEMI on ECG					
Number of residents (n)	30	32	29	91	
Standard written test	18 (60.0)	28 (87.5)	25 (86.2)	71 (78.0)	0.01
Simulation center	26 (86.7)	31 (96.9)	27 (93.1)	84 (92.3)	0.35
P-value	0.67^b^	0.71^b^	0.56^b^	0.66^b^	
Critical actions					
Properly managed septic shock	27 (90.0)	32 (100.0)	28 (96.6)	87 (95.6)	0.22
Ordered IV fluids appropriately	29 (96.7)	32 (100.0)	29 (100.0)	90 (98.9)	0.22
Ordered antibiotics appropriately	27 (90.0)	32 (100.0)	28 (96.6)	87 (95.6)	0.22
Acted on STEMI	25 (83.3)	30 (93.8)	27 (93.1)	82 (90.1)	0.21
Activated cardiac catheter lab	12 (40.0)	18 (56.3)	9 (31.0)	39 (42.9)	0.50
Attempts to personally see patient	3 (10.0)	2 (6.3)	8 (27.6)	13 (14.3)	0.06
Asks 2nd MD to see STEMI patient	8 (26.7)	8 (25.0)	8 (27.6)	24 (26.4)	0.94
Properly managed septic shock, identified STEMI, & acted on STEMI	22 (73.3)	30 (93.8)	26 (100.0)	78 (85.7)	0.07

*PGY*, post graduate year; *STEMI*, ST segment elevation myocardial infarction; *ECG*, electrocardiogram; *MD*, medical doctor

a p-value determined by Cochran-Mantel-Haenszel Contingency Table Analysis.

b p-value determined by Repeated Measures Logistic Regression.
